# Impact of fetal brain tissue derived mesenchymal stem cell and fibrin glue on facial nerve crash injury

**DOI:** 10.3906/sag-2004-3

**Published:** 2021-06-28

**Authors:** Ömer BAYIR, Tuğba KARAGÖZ, Ferda ALPASLAN PINARLI, Gülistan Sanem SARIBAŞ, Candan ÖZOĞUL, Kemal KESEROĞLU, Güleser SAYLAM, Emel ÇADALLI TATAR, Sevilay KARAHAN, Bülent ÖCAL, Mehmet Hakan KORKMAZ

**Affiliations:** 1 Department of Otolaryngology Head and Neck Surgery, University of Health Sciences, Dışkapı Yıldırım Beyazıt Training and Research Hospital, Ankara Turkey; 2 Department of Otorhinolaryngology and Head and Neck Surgery, Kaman State Hospital, Kırşehir, Turkey 3Myogen Laboratory and Health Services, Ankara Turkey; 3 Myogen Laboratory and Health Services, Ankara Turkey; 4 Department of Histology and Embryology, Faculty of Medicine, Ahi Evran University, Kırşehir Turkey; 5 Department of Histology and Embryology, Faculty of Medicine, University of Kyrenia, Girne Turkish Republic of Nothern Cyprus; 6 Department of Biostatistics, Faculty of Medicine, Hacettepe University, Ankara Turkey; 7 Department of Otolaryngology Head and Neck Surgery, Faculty of Medicine, Yıldırım Beyazıt University, Ankara Turkey

**Keywords:** Facial nerve, facial nerve palsy, trauma, stem cell implantation, fetal brain tissue derived mesenchymal stem cells

## Abstract

**Background/aim:**

To evaluate the clinical and histopathological effects of fetal brain tissue derived mesenchymal stem cells (FBTMSC) and fibrin glue (FG) on the facial nerve (FN) regeneration in rats with traumatic FN injury.

**Materials and methods:**

Twenty-eight Sprague Dawley rats were included in the study and divided into 4 groups. Traumatic FN injury (FP) was created by a surgical clamp compression to the main trunk of left FN in all groups. In the control group (group 1) no treatment was applied, in group 2 (FBTMSC group) 2 × 106 FBTMSC was injected, in group 3 (FG group) only FG was applied, in group 4 (FBTMSC and FG groups) both FBTMSC and FG were applied to the injured section of the nerve. The FN functions were evaluated clinically, immediately after the procedure and at 3rd, 5th, and 8th weeks postoperatively. The FNs of all subjects were excised after the 8th week; then the rats were sacrificed. The presence of stem cells in the injured zone was assessed using bromo-deoxyuridine (BrdU), and apoptosis was determined by the TUNEL method.

**Results:**

After the damage, total FP was observed in all subjects. Statistically significant functional improvement was observed in group 4 compared to all other groups (P < 0.005). TUNEL-positive cell count was statistically significantly higher in the control group than the other groups (P < 0.001). TUNEL-positive cell count was statistically significantly lower in group 4 than the other groups. The proportion of BrdU-stained cells in group 4 (5%) was higher than group 2 (2%).

**Conclusion:**

Clinically and histopathologically FBTMSC applied with FG may play a promising role as a regenerative treatment in posttraumatic FP.

## 1. Introduction

The facial nerve (FN), with its multiple functions, is one of the most sensitive cranial nerves against traumas due to its complex and unique anatomical structure [1]. The paralysis of the facial nerve (FP) causes worsening of the quality of life and consequent social and psychological disorders, owing to loss of many functions and aesthetic appearance in face. Therefore, full recovery of FP is very important.

The most common cause is Bell’s palsy. Traumatic FP that develops due to blunt and penetrating traumas, and iatrogenic causes is the second most common. Infections and tumors are rare causes of FP [1]. Recovery may not be satisfactory after complete axonal injury of FN [2]. In addition, Wallerian degeneration after axonal damage starts to develop within days[3].

Although many methods have been used so far in the rehabilitation of FP, there have been no excellent effective results in functional recovery [4,5]. These methods include observation with or without medical treatment, physical therapy, surgical FN decompression, neurorrhaphy, autologous nerve grafting, and nerve transposition [6,7]. Although the most successful surgical procedures are neurorrhaphy and autologous nerve grafting, these procedures have some limitations including limited donor nerves, nonaesthetic scarring, infection, pain, long surgical times, and inadequate regeneration [2]. If sufficient regeneration does not develop, facial reanimation techniques such as muscle transpositions and static suspension procedures are alternative solutions. However, despite all these techniques, the healing process may result in sequelae [8]. New treatment modalities are still being developed to prevent these results and improve the quality of life of patients.

Stem cell transplantation is a very successful treatment modality using tissue engineering in many different diseases. The last 2 decades of studies have shown the positive effects of various stem cell therapies related to nerve regeneration, in both the central and peripheral nervous system [2,9,10]. It has been shown that the stem cell is differentiated into Schwann cell phenotype and accelerates axonal regeneration and provides better remyelination [11,12]. It has also been shown that; stem cells increase growth factor secretion in peripheral nerve damage and thus augment regeneration with antiinflammatory action, and replace damaged Schwann cells and motor neurons [2,9,13].

There are studies using adult, embryo and fetal stem cells for the regeneration of nerve damage. Many subtypes of these stem cells such as bone marrow, mesenchymal, and neural stem cells have been used for regeneration in peripheral nerve damage [2,9,13,14]. In previous years, animal studies were carried out using many kind of stem cells in the FP that reported good results [15–22]. There is no research in the literature about the effects of fetal brain tissue-oriented mesenchymal stem cells (FBTMSC) on facial nerve damage. 

It has been shown that some biodegradable materials and pharmacological agents may contribute to nerve regeneration. These materials provide a protein rich environment, increase stem cell adhesion and promote axonal growth [2,23]. For this purpose, many different materials and pharmacological agents have been used [9,15,17–20,22,24,25]. Fibrin glue (FG) application alone has been shown in the studies that accelerate nerve regeneration [26,27].

In this study, we aimed to investigate the effects of local administration of rat FBTMSC together with FG, on functional and histological recovery of crush injury of FP in rats.

## 2. Materials and methods

### 2.1. Study population and design

A total of 28 adult female Sprague Dawley rats weighing between 250 and 300 g were included in the study. Subjects were hosted in the Experimental Animals Laboratory, during the study, within an environment of 12 h light/12 h dark, temperature of 24–26 ° C with 60%–70% moisture, and fed ad libitum. The study was carried out with the approval of the Animal Ethics Board of our hospital (2015/2024). 

The animals were anesthetized and then left facial nerves were identified surgically in all groups. The left facial nerves of all animals were damaged by a direct surgical mosquito compression. Fifteen minutes after the iatrogenic injury to the nerve, different treatment modalities were applied for each group:

1. Control group: no treatment was applied (group 1, n = 7).

2. FBTMSC group *:* 2 × 106 FBTMSC was injected into the damaged zone (group 2, n = 7).

3. FG group: only fibrin glue (FG) (Tisseel VH; Baxter, Vienna, Austria) was applied over the damaged zone (group 3, n = 7).

4. FBTMSC and FG groups: the damaged part was first injected 2 × 106 FBTOMSC and then covered with FG (group 4, n = 7).

Facial functions of the subjects were evaluated immediately after recovery from anesthesia, and then 3, 5 and 8 weeks after surgery. The damaged section of the FN of all subjects was excised at the 8th week for histopathological examinations (TUNEL) and evaluation of stem cell implantation (BrdU). All subjects were then sacrificed.

### 2.2. Detection, identification, and marking of mesenchymal stem cells from fetal brain tissue

**Figure 1:**
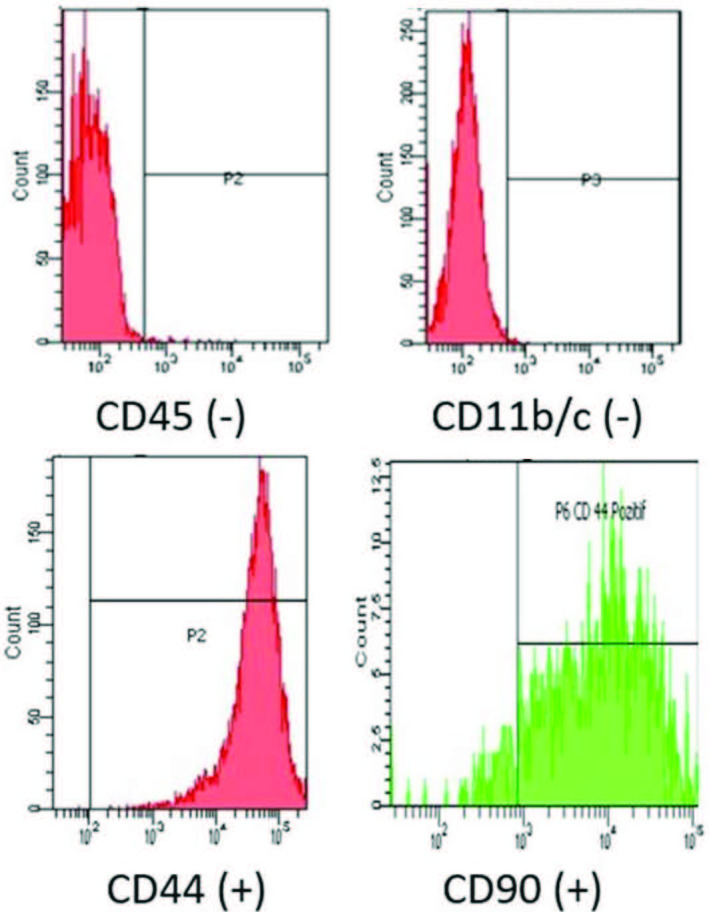
Characterization of FBTMSCs in flow cytometer according to their surface markers (FACSAria III, USA) with: CD11b/c (–), CD45 (–), CD90 (+), CD44 (+).

The brain tissue was removed from the fetus taken from an 18-days pregnant rat under deep anesthesia with xylazine and ketamine in sterile conditions. It was divided into 1 cm3 pieces and seeded in culture dishes with the explant method. Passage of the mesenchymal stem cell cultures in 5% CO2 incubator (20% fetal bovine serum (Lonza, Verviers, Belgium), 2% L-glutamine (Lonza), 1% penicillin, streptomycin, amphotericin (Biological Industries, Beit-Haemek, Israel) and 77% Dulbecco’s modified eagle medium (DMEM-LG, Lonza) was done when the cells filled 80% of the culture dishes by changing the medium once every 3 days. After the second passage, the cells were identified in flow cytometry (FACSAria III, San Jose, CA,USA) according to CD11b/c (BD, USA), CD45 (BD, USA), CD90 (BD, USA), CD44 (BD, USA) surface markers (Figure 1). Cells after the second passage were then differentiated into adipocytes, osteocytes, and chondrocytes by the following differentiation methods. 

**Figure 2:**
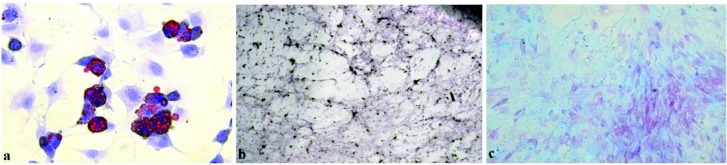
Histochemical staining of adipocyte, osteocyte and chondrocyte transformation representing the transformation of three different germ-layer structures for the characterization of FBTMSCs: a: demonstration of adipocyte differentiation (Oil Red, Diagnostics BioSystem) with the appearance of lipid droplets in the cell to appear red(Leica D50, 10   40). b: demonstration of osteocyte differentiation (Von Kossa, Diagnostic BioSystem) with staining of calcium deposits according to the principle of black staining (Leica D50, 10   10). c: demonstration of chondrocyte differentiation (Alica blue, Diagnostics BioSystem) and hyaluronic acid staining based on blue/purple staining principle (Leica D50, 10   20).

MSCs obtained from the second passage were placed in the culture dishes after the passage and when they covered more than 40% of the container and they were taken to the adipocyte medium [adipocyte differential basal medium and supplement (Gibco, Gaithersburg, MD,USA)]. Replacing the medium on a 3-day basis, dyeing was performed according to the principle that lipid droplets appear red within the cell with Oil Red (Diagnostics BioSystem, Pleasanton, CA, USA) at the end of the 3rd week (Figure 2a).

For differentiation of osteocytes, the osteocyte differentiation basal medium and supplement (Gibco) were used. MSCs from the second passage were planted in the culture dishes after the passage and after covering 40% of the dishes, the medium was changed with osteocyte medium every 3 days. At the end of the 3rd week, staining was performed according to the principle of black staining of calcium deposits with the Von Kossa staining system (Diagnostics BioSystem) (Figure 2b).

MSCs from the second passage were re-passaged, and when they covered 40% of the culture dishes, chondrocyte medium (chondrocyte differential basal medium and supplementation (Gibco) was added for chondrocyte differentiation every 3 days. At the end of the 3rd week, Alcian blue (Diagnostics BioSystem) was used to dye hyaluronic acid based on the blue/purple staining principle (Figure 2c).

Before transplantation, the number and viability of the cells were evaluated with the Countess automated cell counter (Invitrogen, USA) and the cells stained with 10 μL/mL BrdU (bromo-deoxyuridine, 1 mM BrdU in 1 × Dulbecco’s phosphate buffered saline, BD Pharmingen, US) that were prepared at a concentration of 2 × 106 cells/mL. The solution was carefully added and incubated for 2 h. Cells labeled with BrdU were given in a 1cc insulin injector [1cc Dulbecco 1s PBS + 2 × 106 cells (FBTMSC)] to be used in the experiment.

The 4-µm (micrometer) sections were obtained from formalin-fixed, paraffin-embedded tissues and then the tissues were treated with sequential alcohol and deparaffinized with xylen. Immunohistochemical staining was performed with BrdU detection kit (BD-Pharmingen), after treatment with 3% H2O2 followed by washing with PBS. The ratio of BrdU(+) was obtained by counting 100 cells, 3 times in 20 (20 X 10) areas, from the specimens that included facial nerve segments from the rats treated with FBTMSC.

### 2.3. Surgical procedure

After anesthesia with xylazine hydrochloride (3 mg/kg) and ketamine hydrochloride (100 mg/kg), the left infraauricular region was shaved and sterilized to create FN damage. A horizontal incision extending from the postauricular region to the mandibular side was made and the skin and subcutaneous tissues were passed, and the parotid gland was reached. The parotid gland was excised and the FN main trunk was identified at the anterior aspect of the posterior digastric muscle. The nerve damage was created just proximal to the nerve trifurcation using microforceps compression for 30 s. The damage was repeated for another 30 s. The same researcher performed the compression to all subjects. Stem cells were injected into the damaged area, and FG was applied to the same area as well. The surgical area was then properly closed. The subjects were anesthetized 8 weeks after the initial procedure as mentioned above. The FN was reexposed with the same incision, the damaged area was found and the injured nerve segment was resected. Then, FN specimen was prepared and fixated in 4% paraformaldehyde solution. The rats were then sacrificed.

### 2.4. Physical examination evaluation method

Immediately after the initial surgical procedure and at the 3rd, 5th, and 8th weeks postoperatively, the FN motor function examinations of all rats were done according to the standardized scale with observation of vibrissae movement and the standardized scale with observation of blink reflex and eye closure [28].

### 2.5. Histochemical method

The FN tissue specimens obtained from all subjects were first fixated in 10% formaldehyde solution for at least 72 h for light microscopic examination. After fixation, tissue samples were placed in cassettes and washed under stream for 24 h. Tissues were removed from the increased alcohol series (50%, 70%, 80%, 90%, 100%) for removal of water. Afterwards, the tissues were passed through xylene to be transparent and then embedded in paraffin. Four-µm thick sections were cut from prepared paraffin blocks.

### 2.6. TUNEL assay

The TUNEL (terminal deoxynucleotidyl transferase dUTP nick end labeling) method was used to determine apoptosis developed by DNA fragmentation. Millipore Apoptag Plus peroxidase in situ apoptosis detection kit (Millipore, Cat no: S7101, Darmstadt, Germany) was used for this method. Cross sections were incubated at 61 °C. After deparaffinization, the tissues were incubated with 20 μg/mL proteinase K (Millipore, Catalogue no. 21627, Germany) at 37 °C for 25 min. Endogenous peroxidase activity was blocked in tissues with 3% hydrogen peroxide for 15 min. The cross sections were incubated with the equilibration buffer for 5 min at room temperature. Sections were incubated with working strength TdT enzyme solution in a humid environment at 37 °C for 60 min. Then, tissues were incubated for 10 min in stop/wash buffer solution and then incubated with antidigoxin peroxidase solution for 30 min in a humid environment. Subsequent staining with diaminobenzidine was used to identify TUNEL-positive cells. Mayer’s hematoxylin was used as a background stain. Cross sections were evaluated under a light microscope using a computer-supported imaging system to take photographs using the Leica Q Vin 3 program. TUNEL-positive cells were counted at 400 × magnification in random areas containing the nerve bundles in the tissue sections of the subjects.

### 2.7. Statistical analysis

The normal distribution of the variables was analyzed graphically and by the Shapiro–Wilk test. Descriptive data were presented with mean ± standard deviation and median (25th–75th percentiles) values. The Kruskal–Wallis nonparametric analysis of variance was used to compare the skewed data that did not provide parametric test assumptions. Within time differences were analyzed by the Friedman test. In order to determine the different group, a Connover–Dunn pairwise comparison test was performed. Statistical analysis and calculations were performed using IBM SPSS Statistics Version 22.0 (IBM Corp., Armonk, NY, USA) program. In statistical decisions P ≤ 0.05 was accepted as an indicator of significant difference.

## 3. Results

### 3.1. Physical examination evaluation

Left total FP was observed in all rats immediately after the procedure, and at the 3rd, 5th, and 8th weeks blink reflex, eye closure and vibrissae position were compared. According to intra-group comparisons; in all groups, the 5th and 8th week values were found to be statistically significantly better than the postoperative results. According to the comparisons between groups; in all weeks, blink reflex and eye closure degree in group 4 were found to be statistically better than the other groups (Table 1).

**Table 1:** Statistical evaluation of blink reflex and eye closure results.

Groups	After surgery		3rd week		5th week		8th week		P2
	median	25th–75th pctl	median	25th–75th pctl	median	25th–75th pctl	median	25th–75th pctl	
Control (group 1)	1.00	1.00–1.00	1.00	1.00–2.00	3.00	2.00–3.00	3.00	1.00–4.00	0.012*
FBTMSC (group 2)	1.00	1.00–1.00	2.00	1.00–3.00	3.00	2.00–3.00	4.00	3.00–4.00	<0.001*
FG (group 3)	1.00	1.00–1.00	2.00	1.00–2.00	2.00	2.00–3.00	2.00	2.00–4.00	0.001*
FBTMSC and FG (group 4)	1.00	1.00–1.00	3.00	3.00–3.00	4.00	3.00–4.00	5.00	4.00–5.00	<0.001*
P1	1.000		0.002		0.037		0.003		

P1; Kruskal–Wallis test, P2; Friedman test.

The vibrissae monitoring score was found to be the highest in group 4 at the 3rd week evaluation, and there was a difference between group 4 and group 3 only at the 5th week. The results of group 1 and group 3 at the 8th week were significantly lower than that of group 2 and group 4. When compared to the immediate postinjury evaluations; in all groups 5th and 8th week results were better (Table 2).

**Table 2:** Statistical evaluation of Vibrissae clinical observation.

Groups	After surgery		3rd week		5th week		8th week		P2
	Median	25th–75th pctl	Median	25th–75th pctl	Median	25th–75th pctl	Median	25th–75th pctl	
Control (group 1)	1.00	1.00–1.00	2.00	2.00–2.00	3.00	3.00–3.00	3.00	3.00–3.00	<0.001*
FBTMSC (group 2)	1.00	1.00–1.00	2.00	2.00–2.00	3.00	3.00–3.00	4.00	4.00–4.00	<0.001*
FG (group 3)	1.00	1.00–1.00	2.00	1.00–2.00	2.00	2.00–3.00	3.00	2.00–3.00	0.001*
FBTMSC and FG (group 4)	1.00	1.00–1.00	3.00	3.00–4.00	3.00	3.00–4.00	5.00	4.00–5.00	<0.001*
P1	1.000		0.001		0.014		<0.001		

P1; Kruskal–Wallis test, P2; Friedman test.

### 3.2. TUNEL

**Figure 3:**
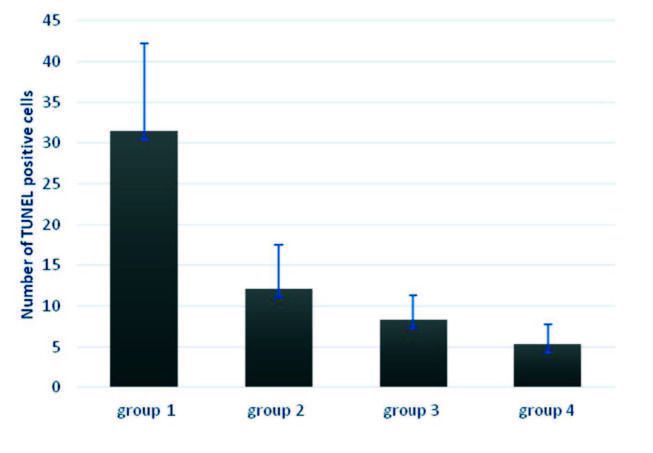
Quantitative summary of TUNEL-positive cell count for all groups.

**Figure 4:**
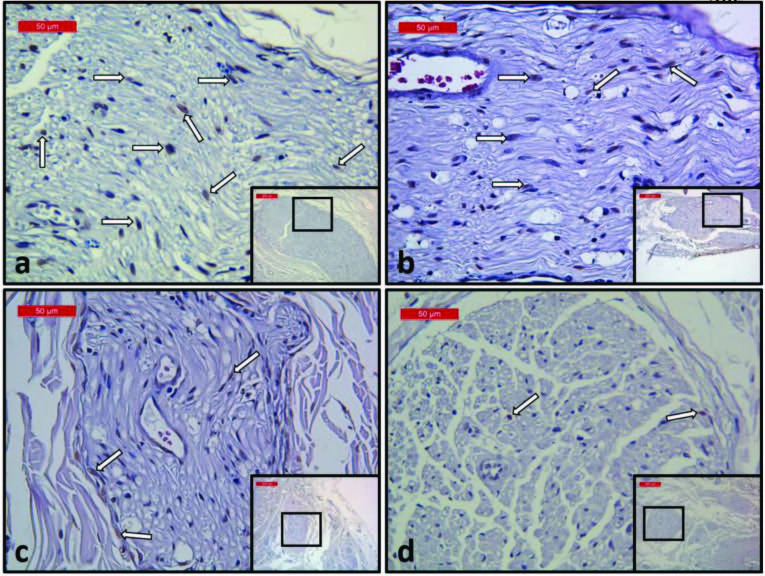
Photomicrographs representing TUNEL-positive staining on the nerve tissues of all the groups (DAB, hematoxylin) a: the highest number of positive cells in the control group; b: reduced number of positive cells in the FBTMSC group; c: reduced number of positive cells in the FG group; d: minimum number of positive cells in the FBTMSC and FG groups.

TUNEL-positive cell values in all groups and were obtained as 31.42 ± 10.84; 12.06 ± 5.44; 8.28 ± 3.04 and 5.33 ± 2.44 respectively. The TUNEL-positive cell count of group 1 was significantly higher than the treatment groups (Figures 3 and 4a). The number of TUNEL-positive cells decreased in all treatment groups compared to the control group (P < 0.001) (Figure 3). The difference between group 2 (Figure 4b) and group 4 was found to be statistically significant (P < 0.001). Similarly, there was a statistically difference between group 3 (Figure 4c) and group 4 (Figure 4d) (P < 0.041). However, there was no significant difference between the groups 2 and 3 (P = 0.167) (Figures 3 and 4).

### 3.3. BrdU staining results

**Figure 5:**
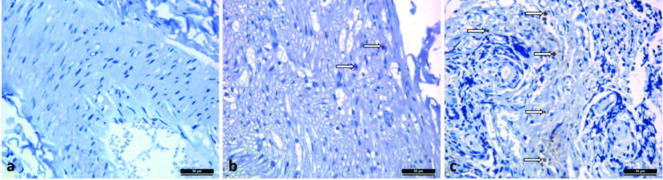
Demonstration of BrR1-labeled MSCs in tissues by immunohistochemical staining (brown nucleated cells). a: control group/group 1 [BrdU (–)]. b: FBTMSC group/group 2 [BrdU (+)]. c: FBTMSC and FG groups/group 4 [BrdU (+)] Leica DM 4000 (Wetzlar, Germany).

BrdU staining results of BrdU detection kit (BD-Pharmingen) showed the percentage of BrdU(+) (Prepared by BrdU and obtained by counting 100 cells 3 times in 20 X 10) in 20 (20 X 10) area of the facial nerve traction obtained from rats. No staining was observed in group 1 (Figure 5a) whereas 2% was found in group 2 (Figure 5b), as well as 5% in group 4 (Figure 5c). 

## 4. Discussion

Although peripheral nerves are capable of self-healing after traumas; regeneration and functional improvements after severe damages may not be satisfactory [18]. Any new method or treatment modality to accelerate regeneration and provide full functional recovery in traumatic nerve paralysis, is invaluable. Recent advances in tissue engineering and stem cell transplantations with different cell differentiation capacities give a new perspective to this field. It has been shown in several studies that, stem cells, can accelerate the regeneration of the damaged nerves by producing adhesion molecules, neurotrophins and growth factors or by replacing Schwann cells or even by replacing the neurons [2,9,13,14]. It is reported that Schwann cell plays a major role in peripheral nerve regeneration and can be used in nerve injuries since the differentiation of the stem cell to the Schwann cell was determined [11]. Mesenchymal stem/stromal cells have been shown to have antiinflammatory effects; by expressing IL-1 receptor antagonists and also have negative feedback effects on proinflammatory cytokines [29]. This is another feature that will explain the positive effects of stem cell therapy on nerve regeneration. Similarly, another study has shown that mesenchymal stem cell accelerates axonal growth by neurotrophic and proangiogenic activity [30].

The effectiveness of stem cell technology in different peripheral neural damages has been shown in studies. In one study, local mesenchymal stem cell injection in the damaged area has been shown to accelerate recovery in recurrent laryngeal nerve injury [31]. In their study, Lerner et al. found no significant difference with the control group in terms of functional recovery in the recurrent laryngeal nerve injury with intravenous mesenchymal stem cell therapy; but the control group of this study also gained normal functions in less time than expected [32]. In experimental animal models, stem cells have been shown to provide nerve regeneration in sciatic nerve injury through antiinflammatory effects, growth factor secretion, Schwann cell or motor neuron differentiation [13]. Matthes et al. showed that intravenous transplantation of mesenchymal stromal cells to rats with sciatic nerve injury contributed to peripheral nerve regeneration clinically and histologically with footprint analysis [33]. In this study, 21 days after intravenous transplantation of mesenchymal stromal cells, histological examination of the damaged nerve showed that these cells were present in the area where nerve damage was present and functional recovery was superior as well. The study of Yamamoto et al. is based on the potential neurotrophic and neuro-differentiated feature of mesenchymal stem cells. Dental pulp stem cell effect was compared with autograft and collagen scaffolds on the damaged sciatic nerve in rats and regeneration in the stem cell transected group was superior to other groups [34]. Considering the high likelihood of sequelae in severe traumatic FP and medical treatment for sequelae may not be sufficient, it is mandatory to develop a new treatment alternative. Unlike other skeletal muscles, the atrophy of the mimic muscles develops many years after nerve paralysis, but even without atrophy, FP causes a high level of psychological stress. There are some experimental stem cell studies in the literature for facial nerve injury.

In posttraumatic FP in humans, facial decompression was performed and a positive result was obtained in a study, in which bone marrow mononuclear stem cells were also applied to the damaged area [7]. Wang et al. compared the bone marrow mesenchymal stem cell application with Schwann-like mesenchymal stem cells in rabbits with facial nerve damage, and concluded that the Schwann-like mesenchymal stem cell gave more positive results in axon regeneration and remyelination [15]. Batioglu–Karaaltin et al. showed the efficacy of human olfactory stem cell in nerve regeneration in facial nerve damage they formed in rats [16]. Zhang et al. in a model of facial nerve damage they created in rabbits, used neural stem cells supplemented with hyaluronic acid, collagen and neurotrophin-3 and determined that this might be an alternative modality in the management of the treatment of peripheral nerve defects [17]. Cho et al. in a model of facial nerve injury that they formed in guinea pigs, used neural mesenchymal stem cells together with platelet-rich plasma. In this study, the authors concluded that only stem cell administration is not effective but it promotes nerve regeneration of the stem cell used with platelet-rich plasma [18]. Ma et al. used neural stem cells supplemented with fibroblast growth factor on collagen scaffold in facial nerve injury and observed significant neural proliferation and functional recovery [19]. Sasaki et al. applied poly-DL-lactide-co-glycole tubes carrying dental pulp cells on damaged rat facial nerves and observed remyelination and regeneration in nerves [20]. Satar et al. in their study, created damage to facial nerve branches in rats and applied mesenchymal stem cells [21]. They stated that mesenchymal stem cell had positive effects on histopathological damage on facial nerve branches. Shi et al. applied neural stem cells composed of glia-derived neurotrophic factor, exfoliated polyglycolic acid on damaged rat facial nerves and observed increased physiological, histochemical, and histopathological regeneration [22].

Strategically, the first important point in stem cell therapy is to define which cells can be used in what kind of pathologies. The second important point is the preparation of such cells in a manner which will not harm the patient, and the third important point is the proper placement of these cells. There are various opinions about the superiority of local or systemic administrations in stem cell transplantation [15,35]. Akiyama et al. investigated the effects of intravenous bone marrow cells on demyelinated spinal cord [36]. However, the desired effect in this study was less than expected probably due to the systemic distribution of the given cells. In the studies, stem cells for peripheral nerve injuries have been generally applied locally. For this reason, in our study local injection was performed because of our expectation that it will have a more local effect, which was also thought to be augmented with FG.

FG, with its fibrin-like effect that plays a role in coagulation, has been shown to be as effective as microsurgery on peripheral nerve injury [26]. In an in vivo rat model, it has been shown that regeneration is accelerated by FG in sciatic nerve injury [27]. In another study, growth factor added to FG was shown to increase the expression of p75NTR in peripheral nerve regeneration in Schwann cells [28]. It was stated that FG formed a 3-D matrix for nerve regeneration and its application was easy especially with stem cells. In our study, FG was used to provide stabilization of stem cell in the damaged area, and this 3-D environment was considered to be suitable for neurogenesis [37]. According to the results of our study, both functional and TUNEL outcomes were better in the FG group compared to the control group. The group 4, in which FG was administered with stem cells, yielded the best functional and TUNEL results compared to the other groups.

In the TUNEL test, the highest rate of apoptosis occurred in control group. The second highest rate was observed in group 2. It was considered to be relevant to the fact that FBTMSCs were destroyed very quickly due to high cytotoxic cytokine release in the damaged area. The low incidence of apoptotic cells and the high rate of BrdU(+) in the stem cells group where FBTMSCs were given with fibrin glue, could be interpreted as FG protected the stem cells from the cytokine release and contributed to tissue reparation by keeping these cells in the damaged area. 

In our study, we determined that FBTMSC applied with FG provided the most appropriate microenvironment for nerve regeneration. Our clinical observation results showed that the group reaching the highest functional results was group 4. Although, only stem cell and FG groups also had better results compared with the control group, the expected clinical and histopathological effects were not as fast. At the end of the 8th week, significant clinical improvement was observed in all groups compared to immediate results after the surgical injury. This result could be attributed to the fact that the damage was a crush type rather than a full nerve cut.

In conclusion, in this study, it has been shown both clinically and histopathologically that FBTMSC combined with FG local application might play a promising role as a form of adjunctive regenerative therapy in traumatic facial paralysis. In this context, it is thought that these results will provide a significant contribution to the literature, as well as satisfactory results for patients when they enter clinical practice. However, these results need to be confirmed in a larger population and by human studies. We think that our study will provide a basis for human stem cell studies to provide functional clinical improvement in traumatic FP.

## Financial disclosure

The authors have no conflicting financial interest.
